# Natural compounds in the fight against *Staphylococcus aureus* biofilms: a review of antibiofilm strategies

**DOI:** 10.3389/fphar.2024.1491363

**Published:** 2024-11-20

**Authors:** Milad Kashi, Milad Noei, Zahra Chegini, Aref Shariati

**Affiliations:** ^1^ Student Research Committee, Arak University of Medical Sciences, Arak, Iran; ^2^ Department of Genetics, Faculty of Advanced Science and Technology, Tehran Medical Sciences, Islamic Azad University, Tehran, Iran; ^3^ Department of Microbiology, School of Medicine, Hamadan University of Medical Sciences, Hamadan, Iran; ^4^ Infectious Diseases Research Center (IDRC), Arak University of Medical Sciences, Arak, Iran

**Keywords:** *S. aureus*, biofilm, natural compounds, curcumin, cinnamaldehyde, carvacrol, eugenol, thymol

## Abstract

*Staphylococcus aureus* is an important pathogen due to its ability to form strong biofilms and antibiotic resistance. Biofilms play an important role in bacterial survival against the host immune system and antibiotics. Natural compounds (NCs) have diverse bioactive properties with a low probability of resistance, making them promising candidates for biofilm control. NC such as curcumin, cinnamaldehyde, carvacrol, eugenol, thymol, citral, linalool, 1,8-cineole, pinene, cymene, terpineol, quercetin, and limonene have been widely utilized for the inhibition and destruction of *S. aureus* biofilms. NCs influence biofilm formation through several procedures. Some of the antibiofilm mechanisms of NCs are direct bactericidal effect, disrupting the quorum sensing system, preventing bacteria from aggregation and attachment to surfaces, reducing the microbial surface components recognizing adhesive matrix molecules (MSCRAMMs), interfering with sortase A enzyme, and altering the expression of biofilm-associated genes such as icaADBC, agr, and *sarA*. Furthermore, these compounds affect extracellular polymeric substances (EPS) and their components, such as polysaccharide intercellular adhesin (PIA) and eDNA. However, some disadvantages, such as low water solubility and bioavailability, limit their clinical usage. Therefore, scientists have considered using nanotechnology and drug platforms to improve NC’s efficacy. Some NC, such as thymol and curcumin, can also enhance photodynamic therapy against *S. aurous* biofilm community. This article evaluates the anti-biofilm potential of NC, their mechanisms of action against *S. aureus* biofilms, and various aspects of their application.

## Introduction

*Staphylococcus aureus* is a bacterium that forms biofilms widely linked to infections acquired in community and hospital settings (Mastoor et al., 2022). The bacterium’s capacity to build biofilms restricts the effectiveness of antimicrobial drugs, heightening the infection’s severity and potentially exacerbating the disease’s consequences (e.g., cystic fibrosis), presenting a significant clinical obstacle (Ramasamy et al., 2017b).

The ability of *S. aureus* to attach firmly to both natural and abiotic surfaces is attributed to the presence of proteins that facilitate adhesion to host tissues and surfaces. As a result, it produces biofilms that are both mechanically and chemically resilient (Ramasamy et al., 2017a). A key characteristic of this bacterium is its high concentration of microbial adhesion molecules, referred to as Microbial Surface Component Recognizing Adhesive Matrix Molecules (MSCRAMMs). Intracellular adhesion (IcaA), clumping factors A and B (ClfA and ClfB), collagen-binding adhesion (cna), fibronectin-binding proteins (fnb), and other similar proteins are types of adhesion proteins (Simpson et al., 2004). Notably, while several factors affect the formation of biofilms in *S. aureus*, polysaccharide intercellular adhesins (PIA) expressed by the ica operon have the main impact (Mastoor et al., 2022).

A biofilm is a complex network of closely packed, membrane-like structures created by bacteria that attach to a surface and release a matrix of polysaccharides, fibrin, lipid proteins, and other substances (Xu et al., 2022). Intricate aggregation of extracellular polymers on the biofilm surface results in a complex and organized overall structure that successfully safeguards the stability of the biofilm on the carrier surface. Full eradication of biofilm using conventional methods is often challenging (Srinivasan et al., 2008). Bacterial biofilms enable survival in hostile conditions and frequently exhibit resistance to drugs and human defenses, therefore playing a role in developing persistent illnesses (Kim Y. et al., 2022). Specifically, avoiding the development of harmful biofilms on food and surfaces, especially those of medical equipment, is immensely significant. Multiple processes contribute to the antimicrobial resistance of biofilms, including decreased antibiotic penetration, varying growth rates of bacterial cells, nutritional gradients within the biofilm, and the existence of latent variations (persister phenomena) that are highly resistant to antibiotics. The presence of antibiotics triggers additional mechanisms contributing to the antimicrobial resistance of biofilm. These mechanisms include the production of unique antibiotic-resistance genes specific to biofilm and mutational processes (Kot et al., 2019).

In addition to being resistant to β-lactam antibiotics, methicillin-resistant *S. aure*us (MRSA) strains frequently exhibit resistance to other widely used antibiotic groups, including aminoglycosides, fluoroquinolones, macrolides, tetracycline, and chloramphenicol (Kot et al., 2020). The constrained therapeutic alternatives for MRSA infections lead to elevated mortality rates and escalated budgetary burdens. Consequently, novel approaches, such as nanoparticles (NPs), bacteriophages, enzymes, and natural compounds, have garnered more interest. Natural compounds, such as botanical extracts, oils, and their derived chemicals, have demonstrated efficacy against various microorganisms and have been employed to fight against diseases and infections (Mastoor et al., 2022; Kargaran et al., 2024). A diverse range of secondary metabolites, primarily phenols or their oxygen-substituted derivatives, created by several medicinal plants exhibit a broad spectrum of antibacterial properties (Nostro et al., 2015).

Recent studies have shown that certain natural chemicals, including curcumin, cinnamaldehyde, eugenol, carvacrol, and thymol, not only prevent the production of biofilms but also remove fully developed biofilm formations (Doke et al., 2014; Rangel et al., 2018). Moreover, the concurrent administration of antibacterial medications and various natural compounds can serve as a highly efficient approach to addressing prevalent bacterial infections owing to its heightened potency and efficacy, diminished drug toxicity, optimized dosages, and decreased probability of acquiring resistance strains (Ushimaru et al., 2012). Therefore, this study focuses on the interactions between natural compounds and biofilm communities of *S. aureus*, as well as different pharmacological platforms utilized to enhance the effectiveness of natural compounds against this bacterial biofilm community.

## Carvacrol

Carvacrol, scientifically also referred to as 2-methyl-5-(1-methyl ethyl)-phenol, is a monoterpene phenol found in the essential oils of several *Lamiaceae* species such as *Thymus, Origanum*, *Thymbra*, Satureja, and *Coridothymus*. It has been determined that *Origanum vulgare* contains the greatest quantity of carvacrol (Baser, 2008; Aprotosoaie et al., 2019). This compound is categorized as Generally Recognized as Safe (GRAS) by the U.S. Food and Drug Administration (FDA), and it is used as a flavoring agent in sweets, beverages, and chewing gum (Center for Food and Applied, 2006; Burdock, 2009). The broad-spectrum antibacterial activity and biofilm inhabitation capacity of carvacrol have been extensively investigated (Dorman and Deans, 2000; Inouye et al., 2001; Burt, 2004; Nostro et al., 2015). In this regard, recently published studies have demonstrated the anti-biofilm effect of carvacrol against *S. aureus* (Nostro et al., 2007; Burt et al., 2014; García-Salinas et al., 2018; Peng et al., 2018; Mouwakeh et al., 2019; Kostoglou et al., 2020; Walczak et al., 2021; Li et al., 2022b). For example, in one study, 4–8 μg/mL of carvacrol inhibited *S. aureus* biofilm formation (Peng et al., 2023).

Carvacrol interacts with the lipid bilayer of the bacterial cytoplasmic membrane, leading to a disruption of its integrity, collapse of the proton motive force, extrusion of cellular material, and a reduction in energy metabolism that affects genetic material synthesis (Ben Arfa et al., 2006; García-Salinas et al., 2018; Martínez et al., 2021). Increased membrane damage may hinder the early bacterial attachment phase and disrupt the normal formation of biofilms (Nostro et al., 2012a; Nostro et al., 2012b; Kerekes et al., 2013). In addition, the polar groups present in carvacrol minimize the contact angle values of the material, resulting in a reduction in surface hydrophobicity. This phenomenon may subsequently impact the early stage of bacterial adhesion and undermine biofilms’ typical formation. An alternative hypothesis is that the existence of these molecules on the surface decreased the available space for bacterial invasion (Nostro et al., 2012b). For example, a study found that adding carvacrol and curcumin improved the properties of Poly (Butylene Succinate)-based films. The films displayed significant antibiofilm activity and reduced biofilm formation by 8.22%–87.91%. Due to these properties, the authors suggested that these films can be used in food packaging, medical and pharmaceutical products, and related applications (Łopusiewicz et al., 2021).

**FIGURE 1:**
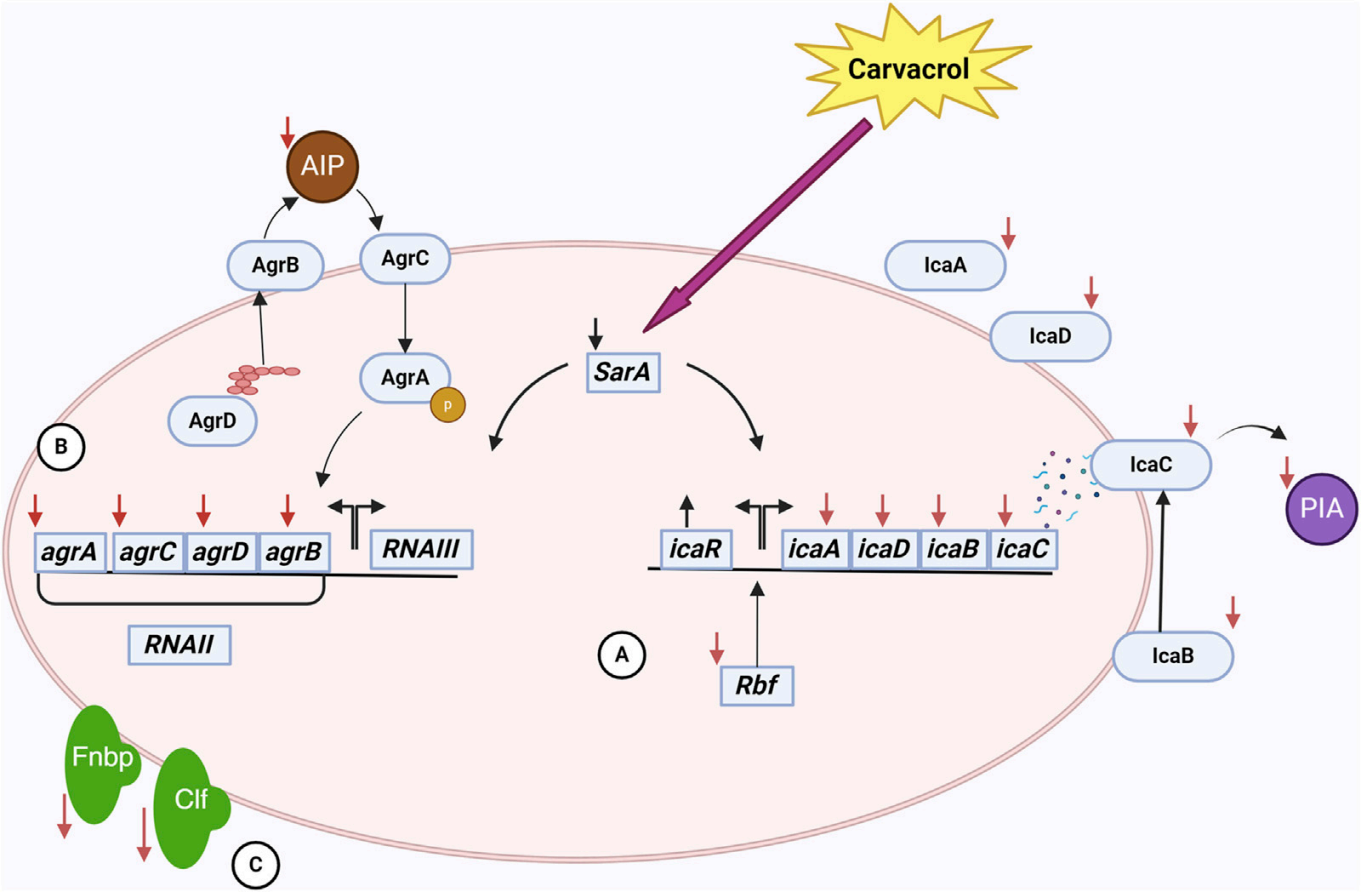
The interactions of carvacrol and *S. aureus* cell in a biofilm community. Carvacrol decreases the expression of the *sarA* gene. This gene affects the ica operon and the agr system. **(A)** By reducing the expression of icaADBC operon and increasing the expression of *icaR* following the use of carvacrol, the expression of proteins affecting the production, processing, and release of PIA/PNAG, an essential component of EPS, occurs. Also, reducing the expression of *rbf* by carvacrol has a negative effect on the ica operon. As a result, EPS and, subsequently, biofilm formation is affected. **(B)** Decreased expression of agr system genes following carvacrol treatment affects the function of the quorum sensing system. In this system, the production, processing, and release of AIP are done by the proteins of this system, and as a result of these changes, the production of AIP decreases. As a result, the communication of cells with each other is disturbed, adversely affecting biofilm formation. **(C)** Also, using carvacrol decreases the expression of *fnbA* and *fnbB* genes. It disrupts the function of ClfB, which results in a decrease in the attachment of bacteria to tissues and disruption in the early stages of biofilm formation.

The biofilm-reducing potency of carvacrol is not necessarily correlated with its biocidal properties. Experimental evidence has demonstrated that carvacrol can impede biofilms’ development without diminishing cell survival. Actually, carvacrol may involve something opposite to the immediate eradication of bacteria (Kachur and Suntres, 2020). It was hypothesized that carvacrol affects the gene coding for quorum sensing (QS). An essential set of regulatory genes involved in biofilm development include *sarA*, *agrA*, and *agrB*. AgrA and AgrB are the primary regulatory molecules of the QS system. Inhibiting their signaling impacts the maturation phase of the biofilm (Burt et al., 2014; Peng et al., 2023). The accessory gene regulator (agr) regulates the QS mechanism and the pathways involved in synthesizing the exopolysaccharide matrix. At sub-inhibitory concentration, carvacrol produced inhibitory effects on the expression of *sarA* and *agrA* (Figure1) (Valliammai et al., 2020b; Li et al., 2023; Martínez et al., 2023; Peng et al., 2023). By regulating *agrA*, carvacrol disrupts QS signaling and subsequently influences biofilm matrix synthesis (Martínez et al., 2023). As a global regulator of biofilm formation, staphylococcal accessory regulator A (SarA) upregulates *ica* operon expression and promotes biofilm development by binding to the *ica* promoter (Tormo María et al., 2005). The *icaADBC* operon encodes PIA, also known as poly-N-acetylglucosamine (PNAG), which is a significant component of the biofilm matrix in *S. aureus* (Jefferson et al., 2004). PIA/PNAG primary role is to facilitate intercellular aggregation, enhance bacterial attachment to the carrier surface, and enable immune evasion, therefore becoming the determining element in the adhesive aggregation stage of the biofilm (Peng et al., 2023).

In addition to PIA/PNAG, SarA also controls adhesion proteins FnbA and FnbB (Fibronectin-binding proteins A and B), which are essential for the attachment of bacterial cells (Brahma et al., 2019; Kot et al., 2019; Li et al., 2019; Valliammai et al., 2020b). As a consequence of the downregulation of SarA, the levels of *icaA*, *icaD*, *fnbA*, and *fnbB* were likewise reduced by the administration of carvacrol (Selvaraj et al., 2020; Alfaiz, 2021; Uc-Cachón et al., 2024). Moreover, carvacrol interacts with SarA through anionic bonding, subsequently affecting biofilm matrix synthesis (Selvaraj et al., 2020). Carvacrol also binds to clumping factor B (ClfB) with high affinity (Alfaiz, 2021). ClfB is an *S. aureus* protein that plays a crucial role in biofilm formation by adhering to host tissues through binding to ligands such as fibrinogen, cytokeratin 10, and other proteins. It can also act to mediate bacterial aggregation and thus enhance the ability of the bacteria to form structured biofilm. Therefore, carvacrol disrupts biofilm formation in this manner (Wertheim et al., 2008; Abraham and Jefferson, 2012).

Additionally, carvacrol negatively regulates the expression of the *rbf* gene (Martínez et al., 2023). The *rbf* gene enhances biofilm formation by stimulating the expression of the *icaADBC* operon, which subsequently leads to increased production of PIA/PNAG (Cue et al., 2009; Cue et al., 2012). Accordingly, by downregulating the *rbf* gene, carvacrol affects biofilm formation. An interesting result of the upregulation of *icaR* by carvacrol is that this gene encodes a transcriptional repressor that decreases the expression of the *icaADBC* operon, resulting in the suppression of PIA synthesis (Peng et al., 2023). By inhibiting the expression of *icaA*, *icaB*, *icaD*, *icaC*, *sarA*, *fnbA*, *fnbB*, *rbf,* and *agrA*, and upregulate *icaR*, carvacrol diminishes PIA/PNAG production, impedes bacterial adhesion, affects bacterial morphology, disrupts QS, and ultimately destabilizes the biofilm (Peng et al., 2023).

In the end, it is noteworthy to mention that cell death and decreased bacterial density leads to the reduction in the expression of QS activation of genes (Gonçalves et al., 2012; Espina et al., 2015; Gobin et al., 2022). Due to the lower initial bacterial counts, the concentration of autoinducers (small molecules secreted by bacteria) decreased. For activation of the QS response, the concentration of autoinducers exceeded a requisite threshold (Karatan and Watnick, 2009). Carvacrol significantly reduced the Autoinducer-2 (AI-2) of *S. aureus* biofilms. This inhibition of AI-2 activity helps reduce biofilm formation and bacterial virulence in *S. aureus* (Li et al., 2020).

The stability of biofilms is attributed to the presence of a matrix composed of extracellular polymeric substances (EPS) generated by bacteria. EPS are the primary constituents of bacterial biofilms and consist of polysaccharides, proteins, and nucleic acids (Krogsgård Nielsen et al., 2017; Nagaraj et al., 2017). EPS reduction may impact the biofilm’s structure and trigger bacterial susceptibility to external stimuli (Selvaraj et al., 2021). Carvacrol has been found to interfere with the synthesis of EPS, making the bacterial community more exposed to environmental threats (Li et al., 2020; Selvaraj et al., 2020). PIA/PNAG is one of the important components of EPS, and as previously mentioned, carvacrol can inhibit PIA/PNAG synthesis (Peng et al., 2023). Another component of EPS is extracellular DNA (eDNA); carvacrol can also reduce the production of eDNA (Li et al., 2023). Additionally, carvacrol inhibits the formation of biofilms by effects on membrane lipids, therefore preventing the buildup of proteins and stopping the microcolony stage (Knowles et al., 2005; Nostro et al., 2007; Miladi et al., 2016; Kasthuri et al., 2022). Furthermore, carvacrol reduces the synthesis of *S. aureus* slime (Sethupathy et al., 2017; Kannappan et al., 2019; Selvaraj et al., 2020). It is important to note that slime synthesis is crucial in biofilm formation (Daniela et al., 2014). Due to its relatively hydrophilic nature, carvacrol exhibits the ability to penetrate through biofilms, which alters their physical stability and destroys the enclosed bacteria (Ben Arfa et al., 2006; Nostro et al., 2012a; Suntres et al., 2015; Li et al., 2020). These results prove that carvacrol can disrupt the biofilm matrix and strengthen the bacterial removal process (Kasthuri et al., 2022).

Factors such as instability, volatility, and low water solubility might reduce the antibacterial effectiveness of essential oils and their components. Additionally, the direct use of carvacrol still faces restrictions (Hyldgaard et al., 2012; Scaffaro et al., 2018). To this end, novel approaches have been devised to generate active systems capable of enhancing the stability and extending the biological efficacy of carvacrol (Nostro et al., 2015; Scaffaro et al., 2018; Ayres Cacciatore et al., 2020; Cui et al., 2024). A practical approach to address this problem is the utilization of liposomes. Liposomes are sphere-shaped colloidal entities with phospholipid bilayer membranes and an interior aqueous compartment. They can encapsulate and regulate vital oil components’ release, enhancing stability and facilitating their biological effects (Desai et al., 2012; Cui et al., 2016a; Cui et al., 2016b). In a study, carvacrol and its isomer, thymol, were encapsulated in liposomes and examined against *S. aureus* and *Salmonella enterica* (Engel et al., 2017). The data obtained indicate a reduced release rate of encapsulated thymol/carvacrol. Short-term therapies with free carvacrol and thymol may be more effective in managing bacterial populations, particularly against *S. aureus*. However, due to their long-lasting antibacterial effects, encapsulated antimicrobials should be considered for disinfecting surfaces and equipment and using them as food preservatives (Pan et al., 2014; Cui et al., 2016a; Engel et al., 2017). In another study, carvacrol was incorporated into electrospun membranes of poly (lactic acid) (PLA) (Scaffaro et al., 2018). The progressive liberation of carvacrol from PLA membranes demonstrated substantial antibacterial efficacy over 144 h, reducing biofilm formation by 92%–96% and 88%–95% for *S. aureus* and *Candida albicans* in single and mixed cultures. Furthermore, a significant reduction in the number of cells, biomass, metabolic activity, and vitality of biofilms formed after 24 and 48 h was shown (Scaffaro et al., 2018). Therefore, as mentioned, the findings of recently published studies highlighted the potential of nanobiotechnology, specifically electrospun nanofibrous membranes, as a viable delivery system for carvacrol. This technology offers an ecological alternative in developing novel antibiofilm strategies and shows promise as an agent for controlling infections associated with *S. aureus* biofilms. Briefly, carvacrol disrupts biofilm formation through different mechanisms, including interference with QS, membrane disruption, inhibition of bacterial adhesion, matrix penetration, inhibition of EPS production, and gene expression changes. Therefore, these manifold effects make carvacrol a potent agent against *S. aureus* biofilms.

## Curcumin

Curcumin is an orange-yellow pigment found in the rhizome of *Curcuma longa* (Borra et al., 2014). Curcumin exhibits a wide range of therapeutic effects, including antimicrobial, and antiseptic activities (Prakash et al., 2011; Kunnumakkara et al., 2017; Wang H. et al., 2019). Curcumin has been shown in recent research to effectively suppress the development of biofilms, particularly in Gram-positive bacteria (Moshe et al., 2011; Batista de Andrade Neto et al., 2021; Alqahtani et al., 2024). An *in vitro* study demonstrated that a 100 μg/mL concentration of curcumin successfully inhibits the development of *S. aureus* biofilm (Moshe et al., 2011). Noteworthy, curcumin has the potential to disrupt the structural integrity of the bacterial cell membrane before the initial stages of biofilm development, which include the attachment of cells to a surface, the assembly of cells to form micro colonies, and the maturation of the biofilm into a cohesive structure (Tan et al., 2019; Pamukçu et al., 2022). Additionally, curcumin can interfere with the planktonic cells and further inhibit biofilm reformation (Tan et al., 2019).

The previously published research findings indicated that the curcumin concentration needed to suppress biofilm formation was far lower than the dosage needed to suppress *S. aureus* growth. Accordingly, the authors proposed that the inhibitory effect of curcumin on biofilm formation is attributed to its ability to impede the process of biofilm formation itself rather than its bactericidal properties (Moshe et al., 2011). Therefore, curcumin has shown good potential by targeting bacterial adhesion and preventing biofilm formation. One of the possible mechanisms for this phenomenon is an interaction of curcumin with enzymes necessary for bacterial attachment to the host cells. For example, sortase A, an enzyme essential for the ability to attachment to host tissues, is one of these enzymes. By inhibiting sortase A, cells cannot bind to cell-matrix proteins, such as fibronectin, thus curcumin disrupting the process of adhesion (Park et al., 2005; Loo et al., 2016). Apart from targeting sortase A, the *fnbA* gene and clumping factor A (*clfA*) were downregulated by curcumin (Khaleghian et al., 2023). FnbA and ClfA facilitate bacterial aggregation and adherence to host tissues and surfaces by binding to fibronectin and fibrinogen. This interaction is essential for the initial stages of biofilm development (Lebeaux et al., 2013; Murai et al., 2016). Also, curcumin significantly decreases elastin-binding protein (ebp) expression, which plays a role in the binding of *S. aureus* to the host elastin protein and promotes bacterial attachment and invasion (Targhi et al., 2021). Consequently, curcumin interferes with the attachment of bacteria during biofilm formation.

Besides anti-adhesion activity, curcumin prevented biofilm formation by interfering with EPS synthesis. Effective interaction of curcumin with the biofilm-forming proteins of *S. aureus* results in reduced microbial biomass and generation of EPS, which are crucial for biofilm structure (Akhtar and Khan, 2021; Akhtar et al., 2021; Gao et al., 2023; Sharma et al., 2023). One of those that curcumin interacts with to affect biofilm formation is N-acetylglucosaminyl transferase (IcaD), a protein that produces PIA (Khaleghian et al., 2023). Additionally, a recently published study reported that curcumin downregulated the expression of the *icaADBC* operon genes (*icaA*, *icaB*, *icaC*, *icaD*) (Khaleghian et al., 2023). As mentioned earlier, the *icaADBC* operon encodes proteins and enzymes responsible for PIA synthesis. Therefore, by inhibiting this operon, curcumin interrupts the synthesis of PIA, the attachment of bacteria to each other and surfaces, and thus affects the formation and preservation of biofilm (Vuong et al., 2004). This change makes the biofilm more susceptible to mechanical removal and the action of antimicrobial agents.

Additionally, curcumin can reduce the expression of some genes associated with QS and enhance the proliferation of biofilms (Khaleghian et al., 2023; Sharma et al., 2023). An essential function of the agr system, which consists of AgrB, AgrC, AgrA, and AgrD, is to control virulence factors and biofilm development in *S. aureus* (Bezar et al., 2019). AgrB and AgrD are responsible for producing and processing autoinducible peptide (AIP) (Zhang et al., 2002; Zhang and Ji, 2004). AgrC is a histidine kinase receptor located in the bacterial cell membrane and detects the presence of AIP in the environment (Lina et al., 1998). When AgrC is activated, it phosphorylates AgrA. AgrA then upregulates or downregulates various target genes (Novick et al., 1995). Recent studies showed that curcumin downregulated genes responsible for QS, such as *agrA*, *agrB,* and *agrC* (Khaleghian et al., 2023; Sharma et al., 2023). Studies have shown that the suppression of agr system is important for developing biofilms, whereas the activation of the agr system is crucial for separating biofilms (Boles and Horswill, 2008; Dastgheyb et al., 2015).

Recently published studies have employed the combination of photodynamic therapy (PDT) and curcumin for *S. aureus* biofilm elimination (Table 1). In these studies, curcumin was used as a photosensitizer (PS) and produced reactive oxygen species (ROS) such as superoxide radicals and singlet oxygen molecules (^1^O_2_) (Akhtar et al., 2021; Sharma et al., 2023). ROS oxidizes the biomolecules of microorganisms, resulting in biological damage and decreasing microbial growth, metabolic activities, microbial biomass, and bacterial adhesion ability, and considerable changes in the carbohydrate and protein composition of the extracellular matrix of *S. aureus* (Ribeiro et al., 2022; Sharma et al., 2023). Bacterial cells include ample scavengers, including catalase, peroxidase, and superoxide dismutase, to counteract the bactericidal effects caused by free radicals. However, these scavengers cannot counteract the singlet oxygen molecule, resulting in extensive cell damage when exposed to ^1^O_2_ (Kim et al., 2001; Akhtar et al., 2021). This oxidative stress can damage bacterial cells and inhibit their ability to form and sustain biofilms (Sharma et al., 2023). In addition, the cost of curcumin compared to other photosensitizers is low (Araújo et al., 2014).

**TABLE 1:** Studies that used curcumin-based photodynamic therapy for managing *S. aureus* biofilm.

Year of publication	Study model	Bacteria	Light source	Outcome	References
2018	PDT with CUR	MRSA	LED (450 nm)	The photosensitizer curcumin and blue LED resulted in the reduction of monospecies MRSA biofilms	Araújo et al. (2018)
2020	aPDT (CUR and LED light)	MSSA and MRSA	LED (455 nm)	aPDT significantly reduced biofilm viability for both MSSA and MRSA. MRSA biofilms were generally more resistant to aPDT than MSSA biofilms	Teixeira et al. (2020)
2020	PDT with CUR-silica nanoparticles	*Staphylococcus aureus* and *Pseudomonas aeruginosa*	Laser light (460 nm)	CUR-silica nanoparticles as photosensitizers show a photodynamic inactivation effect against the biofilm form of *S. aureus* and *P. aeruginosa*	Mirzahosseinipour et al. (2020)
2021	CUR-mediated PDT	VRSA	Blue laser (20 J/cm^2^)	aPDT significantly reduced preformed VRSA biofilms	Akhtar et al. (2021)
2021	CUR-aPDT treatment	VRSA	Blue laser light (20 J/cm^2^)	CUR-aPDT-treated VRSA biofilm was nearly completely eradicated. Also, microbial biomass and EPS synthesis were reduced	Akhtar and Khan (2021)
2021	PDT and SPDT with CUR	MSSA	Blue LED light (70 J/cm^2^)	Combining photodynamic and sonodynamic therapy (SPDT) is a promising approach to combat *S. aureus* biofilms	Alves et al. (2021)
2022	AHMSN are used as the carrier for the photosensitizer CUR.	*S. aureus*	Blue LED (450 nm)	Compared with the control group, the number of viable bacteria in the biofilm was reduced by 37.76%–98.20%	Zhao et al. (2022)
2022	Photosensitizer (CUR) and irradiation	MRSA	LED (450 nm)	PDT with CUR significantly reduced the growth of MRSA biofilm. The PDT group showed a notable reduction in bacterial viability	Ribeiro et al. (2022)
2023	PDT with CUR-loaded alginate microfibers	MRSA	Blue LED	When exposed to blue light, CUR-loaded alginate microfibers effectively eradicated the biofilms	Sharma et al. (2023)
2023	PDI, SDI and SPDI with CUR	MSSA	Blue LED (35 J/cm^2^)	All treatments reduced the bacteria’s adhesion ability, cell metabolism, and total biomass and generated ROS. SPDI was more effective in *S. aureus* inactivation	Alves et al. (2023)
2023	aPDT with CUR-loaded micelles and free CUR	MRSA and *Candida albicans*	LED (450nm, 47m/Wcm^2^)	Free CUR and CUR-loaded micelles with blue light decreased the biofilm biomass to 36% and 30% for MRSA and *C. albicans,* respectively	Trigo-Gutierrez et al. (2023)

CUR: curcumin. PDT: photodynamic therapy. MRSA: methicillin-resistant *Staphylococcus aureus*. MSSA: methicillin-susceptible *S. aureus*. VRSA: vancomycin-resistant *S. aureus*. aPDT: antimicrobial Photodynamic Therapy. SPDT: sonophotodynamic therapy. EPS: extracellular polymeric substances. PDI: photodynamic inactivation. SDI: sonodynamic inactivation. ROS: reactive oxygen species. AHMSN: Amino-modified hollow mesoporous silica nanoparticles.

Considerable attempts have been undertaken to enhance the administration of curcumin (Alemi et al., 2018). For example, curcumin loaded on chitosan nanoparticles (CSNP) was used to improve the therapeutic performance of curcumin by increasing its bioavailability (Ma et al., 2020). CSNPs have attracted significant attention as a therapeutic carrier because of their biodegradability, biocompatibility, and freedom from toxicity (Das et al., 2010; Li et al., 2018). A positively charged CSNP can transport curcumin into biofilms and induce its release within the biofilm, affecting the cells therein. However, the inhibitory effect of CSNP-Cur on the biofilm development of *S. aureus* bacteria was somewhat weaker than that of free curcumin. The sustained release of curcumin from CSNP-Cur led to a reduced concentration and diminished antibiofilm action (Ma et al., 2020). However, the diffusion of free curcumin into a preformed biofilm is hindered by the EPS of the biofilm, thereby diminishing the antibiofilm effects of curcumin. Conversely, CSNP-Cur demonstrated superior antibiofilm efficacy compared to free curcumin (Ma et al., 2020). In another study, a niosome was used to encapsulate curcumin to solve the low solubility and stability issue. Niosomal curcumin exhibited a 2-4-fold reduction in multi-drug resistant (MDR) *S. aureus* biofilm relative to free curcumin (Khaleghian et al., 2023).

Additionally, in another study, curcumin was encapsulated in liposomes. In addition to promoting the uptake of this compound in bacterial cells, liposomes provide regulated release of medications. Encapsulating curcumin in liposomes halved its minimum inhibitory concentration (MIC) for *S. aureus* compared to the free form, and antibiofilm activity was observed at lower concentrations (Bhatia et al., 2021). Also, combining curcumin with metal ions can enhance its properties. Curcumin-based metallodrugs increase stabilization and improve curcumin’s bioavailability and solubility (Wanninger et al., 2015). Curcumin conjugating to RuII–polypyridyl complexes [Ru (bpy)2 (cur)] (PF6) showed promising results. Its MIC against *S. aureus* was 1 μg/mL and reduced the biofilm by 48% at 10 × MIC compared to the untreated (Srivastava et al., 2019). The aforementioned findings demonstrate that different drug delivery systems can be employed to augment the effectiveness of curcumin in suppressing biofilm formation. Nevertheless, the available data in this field are currently somewhat restricted, and it is imperative to conduct more comprehensive studies before the clinical application of curcumin-based drug delivery systems.

Curcumin has several ways to disrupt *S. aureus* biofilms, including inhibiting sortase A activity, interfering with attachment, changing bacterial surface properties, interacting with biofilm matrix, and inducing oxidative stress. Together, these factors diminish the ability of *S. aureus* to form or protect its biofilm communities, making them more sensitive to host defenses and traditional antimicrobial treatments.

## Cinnamaldehyde

Cinnamaldehyde is a bioactive compound derived from cinnamon bark, known for its diverse spectrum of effects, including anticancer, antifungal, and antibacterial properties. It has been classified as GRAS by the Flavoring Extract Manufacturers’ Association and has been authorized by the FDA for use in food (Nostro et al., 2012b; Xu et al., 2022). In recent years, scientists have shown interest in utilizing cinnamon and its derivative components, particularly cinnamaldehyde, to suppress *S. aureus* biofilms, in addition to its antibacterial properties (Jia et al., 2011; Nostro et al., 2012b; Zodrow et al., 2012; Budri et al., 2015; Nostro et al., 2015; Campana et al., 2017; García-Salinas et al., 2018; Kot et al., 2018; Mishra et al., 2021; Wang et al., 2021; Kim Y. et al., 2022; Mastoor et al., 2022).

Cinnamaldehyde blocks ATPase and cell-wall biosynthesis and alters membrane structure and integrity to suppress bacteria, yeasts, and filamentous molds (Deng et al., 2018). The results of the Xu et al. study demonstrated that cinnamaldehyde induced the destruction of the cell wall of *S. aureus* and altered the permeability of the cell membrane, leading to the release of potassium ions, alkaline phosphatase, protein, and multiple other compounds (Xu et al., 2022). The results of this study indicated a continual increase in the extracellular potassium ion content in the bacterial solution treated with 1 × MIC of cinnamaldehyde, demonstrating the detrimental effects of this compound on the bacteria (Xu et al., 2022). In line with these findings, another study proposed that the mechanism by which cinnamaldehyde acts may be associated with cell death and/or the deactivation of bacterial virulence factors, regardless of showing high affinity or not to the non-native penicillin-binding protein (PBP2a) responsible for *S. aureus* (Fernandez-Soto et al., 2023).

In addition to the abovementioned research, several studies have investigated the molecular interactions between cinnamaldehyde and *S. aureus* biofilms. An investigation carried out by *Mastoor* et al. revealed that the application of α-methyl-trans-cinnamaldehyde and α-bromo-trans-cinnamaldehyde led to a notable reduction in the expression of *icaA*, *clfA*, and *fnbA* genes in the isolates that were treated. Given the crucial function of *icaA* in biofilm development in *S. aureus*, reducing its gene expression in the treated group could perhaps elucidate the mechanism by which cinnamaldehyde acts against biofilms (Knobloch et al., 2002). In addition, the adhesin proteins ClfA and FnbA, along with other MSCRAMMs, facilitate the early adherence of bacteria to surfaces and are present in all isolates of biofilm-forming *S. aureus*. Hence, the reduction in its expression offers a valuable understanding of the specific mechanism by which the chemical inhibits the development of biofilms (Mastoor et al., 2022). Furthermore, the metabolic activity of *S. aureus* in biofilm was considerably reduced when trans-cinnamaldehyde was present at 1/2 minimum biofilm inhibition concentration (MBIC). Both the weakly and highly adherent strains exhibited reduced expression levels of the genes encoding laminin-binding protein (*eno*), elastin-binding protein (*ebps*), and fibrinogen-binding protein (*fib*) in the presence of trans-cinnamaldehyde at 1/2 MBIC compared to the untreated biofilm. The expression level of *icaA* and *icaD*, which are involved in the manufacture of polysaccharide intercellular adhesion, was more than half lower in the poorly adhering strain with the presence of trans-cinnamaldehyde compared to biofilm without trans-cinnamaldehyde. The findings suggested that trans-cinnamaldehyde effectively inhibits the attachment of MRSA to key components of the extracellular matrix, including elastin and laminin. This inhibition thus hinders the spread of staphylococcal cells and the onset of colonization in host tissue. Thus, the authors postulated that trans-cinnamaldehyde shows potential as an anti-biofilm therapeutic for the treatment of MRSA biofilm-associated infection (Kot et al., 2019).

Finally, the results of the recently published study demonstrated that combining cinnamaldehyde and β-lactam antibiotics can synergistically enhance the activity and sensitivity of clinical MRSA isolates to β-lactam treatment while preventing MRSA biofilm formation. Mechanistic investigations revealed that the potentiating impact of cinnamaldehyde on β-lactams was primarily due to the suppression of *mecA* expression via the targeting of the staphylococcal accessory regulator *sarA*. Cinnamaldehyde alone or in combination with β-lactams reduced the sarA expression and enhanced the SarA protein’s phosphorylation. This process, in turn, hindered the binding of *sarA* to the *mecA* promoter element and suppressed the expression of virulence genes, including those responsible for biofilm formation, α-hemolysin, and adhesin. Impediment of *sarA*–*mecA* interaction disrupted PBP2a production, reducing MRSA resistance to β-lactams. Moreover, cinnamaldehyde completely reinstated the anti-MRSA effects of β-lactam antibiotics in live experimental models of bacteremia and biofilm infections in mice. The authors asserted that cinnamaldehyde functions as a β-lactam adjuvant and can be used as an alternate treatment to address multidrug-resistant MRSA infections (Li J. et al., 2024).

Various drug delivery platforms could be useful in improving cinnamaldehyde efficacy. *Ramasamy* et al. proposed that nanodispersions containing cinnamaldehyde (CNMA) may have exerted their effects by numerous mechanisms, including the inhibition of QS, attachment to cell walls facilitated by the lipophilic character of CNMA, interaction with cytoplasmic contents, release of CNMA, or induction of protein precipitation. Crucially, the activity of cinnamaldehyde attached to the surface of gold nanoparticles (CNMA-GNPs) was significantly higher than that of free CNMA. This finding provides evidence that nanodispersions enhance contact with biofilms. The authors also asserted that the small dimensions of CNMA-GNPs could enable them to penetrate the protective layers of EPS and effectively eliminate bacteria. Moreover, the low pH in biofilm environments can break down nanodispersions and facilitate the persistent release of CNMA (Ramasamy et al., 2017a; Ramasamy et al., 2017b).

Recently published studies reported antibacterial and antibiofilm activity for cinnamaldehyde against *S. aureus*. However, the exact interaction of cinnamaldehyde and this bacterium’s biofilm community is not yet elucidated. Hence, more confirmatory studies are needed in this field, and the usage of nanotechnology to improve the clinical usage of cinnamaldehyde should be considered in future studies.

## Thymol

Thymol, also known as 2-isopropyl-5-methylphenol, is a monoterpene phenol that is widely distributed in several plant species, including *Ocimum gratissimum*, *Thymus vulgaris*, *Thymus ciliates*, *Carum copticum*, *Thymus zygis*, and *Satureja intermedia* (Nagoor Meeran et al., 2017). Thymol is categorized as GRAS by the FDA for use in foods for human consumption or as food additives (Jo et al., 2022). Studies have demonstrated the good antibacterial activity of thymol against various strains of bacteria, including *S. aureus* (Aksoy et al., 2020; Nunes et al., 2021). Furthermore, this compound showed antibiofilm activity against this bacterium in several studies (Nostro et al., 2007; Kifer et al., 2016; Peng et al., 2018; Aksoy et al., 2020; Kostoglou et al., 2020; Jo et al., 2022). For example, in one study, 0.33–0.59 mg/mL of thymol inhibited 90% of *S. aureus* biofilm formation (Kifer et al., 2016).

In a discussion on the antibiofilm activity of thymol, the primary effect is related to its impact on bacterial cell death. Thymol may induce membrane potential depolarization in *S. aureus*, impairing membrane integrity and cellular demise. Consequently, thymol induces an elevation in NADP + levels and a reduction in cytoplasmic NADPH and ATP. Such observation suggests the potential leakage of intracellular constituents and the disturbance of the physiological equilibrium between NADP+ and NADPH. Furthermore, thymol caused a substantial rise in the levels of lipid oxidation throughout the cell membrane (Gómez-Sequeda et al., 2020; Li et al., 2022a). Biofilms treated with thymol showed decreased bacteria and viable cells (Yuan et al., 2020; Jo et al., 2022; Uc-Cachón et al., 2024). In addition, inhibition of bacterial growth and proliferation is achieved by thymol by modification of membrane permeability, which disrupts both protein synthesis and binary fission (Yuan et al., 2020; Walczak et al., 2021). Therefore, with bacterial cell death, the number of cells required to form a biofilm decreases, and thus, the early stages of biofilm formation are disturbed.

As mentioned in the previous part, biofilm formation is initiated by the adhesion of planktonic microorganisms to surfaces and is regarded as a critical phase in the development of biofilms. Thymol significantly reduces the adhesion of *S*. *aureus* and thus suppresses the first stage of biofilm formation (Valliammai et al., 2020a; Jo et al., 2022). Additionally, thymol decreased the expression of *fnbA* and *fnbB* genes, which reduces the adhesion of *S. aureus* to the host tissue (Schröder et al., 2006; Valliammai et al., 2020a).

Biofilms are attached to surfaces by non-specific hydrophobic bonds. These bonds play an important role in the stability and adhesion of biofilms (Rouws et al., 2010; Ali Mirani et al., 2018). Any disruption in these hydrophobic bonds affects the ability of bacteria to attach to surfaces (Wojnicz et al., 2012). In this regard, thymol, as the main compound of *Plectranthus amboinicus*, affected the hydrophobicity of the surface of *S. aureus*, and the surface of bacterial cells became hydrophilic. These changes can affect the adhesion and aggregation of bacteria (Sawant et al., 2022). Therefore, thymol showed anti-adhesion properties that can be used in medical equipment (Bertuola et al., 2018; Valliammai et al., 2021). For example, to control the corrosion of AZ31 Mg alloy as a biodegradable implant and prevent bacterial adhesion, a polymer layer was developed through thymol electro polymerization (TOH). The bacterial adhesion on polyTOH-AZ31 was more than 30-fold smaller than the bare AZ31 alloy. Moreover, PolyTOH-AZ31 increased the effectiveness of antibiotics and inhibited planktonic growth at half of the MIC of the antibiotic (Bertuola et al., 2018).

Thymol can decrease the synthesis of PIA/PNAG as the main components of the EPS matrix in *S. aureus* biofilms (Valliammai et al., 2020a; Yuan et al., 2020; Jo et al., 2022; Uc-Cachón et al., 2024). A recent study indicated that bacteria without PIA/PNAG can initially attach to biomaterials but cannot develop a biofilm at later stages due to a significant decrease in cell-to-cell adhesion (Yuan et al., 2020). Thymol decreased the expression of *sarA* in *S. aureus* and inhibited the expression of other *sarA*-regulated genes, such as *icaA* and *icaD* (Valliammai et al., 2020a; Yuan et al., 2020; Valliammai et al., 2021; Kim B. C. et al., 2022). Notably, these genes significantly affected biofilm formation, and by reducing their expression, the synthesis of PIA, and consequently the formation of biofilm, was affected. In addition, thymol inhibited the release of eDNA, which plays key roles in bacterial adhesion, aggregation, microcolony formation, and biofilm architecture (Yuan et al., 2020). Moreover, thymol downregulated the *cidA* gene in *S. aureus* (Yuan et al., 2020). The holin-like protein (CidA) has been shown to positively increase the release of eDNA during biofilm development (Rice et al., 2007). Besides, thymol, due to its relative hydrophilic nature conferred by the free hydroxyl group, can permeate the polysaccharide matrix of the biofilm and may disrupt it due to its potent inherent antibacterial attributes (Nostro et al., 2007; Miladi et al., 2017; Kostoglou et al., 2020).

Like other natural compounds, the strong antimicrobial effect of thymol is practically limited by its high volatility, insolubility in water, and weak oxidative stability (Amiri et al., 2024). These factors restrict its usage in various practical applications. Therefore, scientists considered the use of new approaches. For instance, thymol loading in chitosan silver nanoparticles (T-C@AgNPs) showed excellent antibacterial activity with MIC = 100 μg/mL against MRSA. Moreover, T-C@AgNPs effectively reduced the attachment of bacteria and downregulated the transcription of the *Coa*, *Eap,* and *SpA* exoprotein genes. The decrease in the mentioned genes indicated a slow binding and a reduction in the coagulation mechanism (Manukumar et al., 2017). Another study has developed thymol-loaded chitosan nanogels (Ty-CsNG) against Gram-negative and Gram-positive MDR bacteria, including *S. aureus*. Ty-CsNG reduced the MIC by 4–6 times compared to free thymol. Moreover, antibiofilm activity and negligible cytotoxicity were observed (Piri-Gharaghie et al., 2022). Utilizing these methods leads to improved pharmacokinetic outcomes for thymol and expands the range of its applications in medicine. Noteworthy, other studies that used drug-platform to improve natural compounds efficacy against *S. aureus* biofilm are presented in Table 2.

**TABLE 2:** Studies utilizing nanoparticles as a delivery platform for various natural compounds to inhibit and eradicate *Staphylococcus aureus* biofilm.

Year of publication	Natural compounds	Drug platforms	Strains	MIC (conc.)	MBIC (conc.)	Outcome	References
2020	Curcumin	Graphene (Gr)-based nano-formulation containing Curcumin and ZnO-NPs	MRSA	31.25–62.5 (µg/mL)	128–512 (µg/mL)	The drag platform inhibited the biofilm more efficiently than monotherapy with GrZnO-NCs and Curcumin alone	Oves et al. (2020)
2020	Limonene	Levofloxacin-loaded limonene-based nanoemulsion	MRSA	3.12 (mg/mL)	½ MIC	Nanoemulsion improved the eradicating efficacy of biofilm. The MIC of the loaded nanoemulgel was two-fold less than that of the drug alone	Mehanna et al. (2020)
2021	Curcumin	Encapsulation of curcumin within a physiological lipid matrix of solid lipid nanoparticles (CSLNs)	*S. aureus*	64 (µg/mL)	512 (µg/mL)	The synthesized nanoparticles demonstrated better penetration and interaction with the biofilm matrix and higher cell uptake	Sandhu et al. (2021)
2021	Gallo-tannin	A natural polyphenol, gallo-tannin, is used to reduce and cap the Fe_2_O_3_ nanoparticles	MDR *S. aureus*, *E. coli* and *Pseudomonas aeruginosa*	500–750 (µg/mL)	½ - 1 MIC	GT-Fe_2_O_3_ exhibited efficient antibacterial properties, inhibited biofilm formation, and disrupted bacterial quorum sensing	Ahmed et al. (2021)
2024	Curcumin	Curcumin-chitosan magnetic nanoparticles (Cur-Chi-MNP)	MRSA and MSSA	4.69 and 75 (μg/mL)	9.38 and 37.5 (μg/mL)	The synthesized nanoparticles showed antimicrobial activity on planktonic cells of *S. aureus* and inhibited the biofilm community	Salazar-Sesatty et al. (2024)
2021	Caffeine	Caff-AuNPs	*S. aureus* KCTC 1916	512 (μg/mL)	256 (μg/mL)	The Caff-AuNPs showed the ability to prevent biofilm formation and disperse mature biofilms	Khan et al. (2021)
2022	Coumaric acid (p-CoA) and gallic acid (GA)	Rhamnolipid (RHL)-coated Fe₃O₄ nanoparticles with p-CoA and GA using polyvinyl alcohol (PVA)	MSSA, MRSA and VRSA	4–32 (μg/mL)	2–16 (μg/mL)	NPs reduced initial adhesion and biofilm formation and downregulated the *icaA* and *icaD* genes	Sharaf et al. (2022)
2024	Rutin	Rut-CS NPs	*S. aureus*	500–1,000 (µg/mL)	NR	½ MIC of Rut-CS NPs effectively inhibited the biofilm formation (22.5%–37.5%)	Esnaashari and Zahmatkesh (2024)

ZnO-NPs, Zinc oxide nanoparticles; MBIC, minimum biofilm inhibitory concentration; MDR, multidrug-resistant; MIC, minimum inhibitory concentration; MSSA, methicillin-susceptible *Staphylococcus aureus*; ROS, reactive oxygen species; MRSA, methicillin-resistant *Staphylococcus aureus*; MBC, minimum bactericidal concentration; VRSA, vancomycin-resistant *Staphylococcus aureus*; NP, nanoparticle; NR, not reported; Rut-CS NPs, Rutin-loaded chitosan nanoparticles; Caff-AuNPs, gold nanoparticles using caffeine.

In the end, thymol can also be used in PDT (Wang Z. et al., 2019; Lu et al., 2021). Thymol acts as a “pro-photosensitizer” and is oxidized to thymoquinone (TQ) and thymohydroquinone (THQ) only in bacteria by blue light. The resultant TQ and THQ act as photosensitizers, enhancing ROS production exponentially and rapidly killing pathogens (Lu et al., 2021). ROS indiscriminately damages cellular components, including lipids, proteins, plasma membranes, and nucleic acids. The 1 × MIC thymol combined with 75 J/cm^2^ or 100 J/cm^2^ blue light could completely remove the viable biofilms of MRSA (Lu et al., 2021). In conventional PDT methods, the photosensitizer enters both bacterial and mammalian cells, generating ROS in both cell types, and posing safety and efficacy challenges. In contrast, thymol as a pro-photosensitizer is only converted to an active photosensitizer in bacteria, and thus, it has higher safety and therapeutic properties. It has the potential for application in topical therapy and biofilm-related treatments, preventing subsequent bacterial invasion or dissemination without causing any adverse effects on the host cells (Lu et al., 2021).

Ultimately, how thymol can suppress the formation of *S. aureus* biofilm includes bacterial death before biofilm formation, inhibiting bacterial movement and attachment, interfering with the structure of the biofilm matrix, and generating reactive oxygen species in photodynamic treatments. However, some drawbacks limit the clinical usage of this natural compound. To this end, scientists should consider using thymol-based drug platforms more when managing bacterial biofilm.

## Eugenol

Eugenol, 4-allyl-2-methoxyphenol, is an odorous oily liquid extracted from specific essential oils, particularly clove and cinnamon, colorless to pale yellow. It has been a flavoring agent in food and cosmetic formulations (Zhang et al., 2018). Empirical investigations have demonstrated that eugenol possesses several potentially advantageous biological characteristics, such as antibacterial, antioxidant, and anti-inflammatory effects (Gill and Holley, 2004; Mohammed and Al-Bayati, 2009; Yadav et al., 2013). Additionally, several studies have demonstrated the eugenol potential for inhibiting and eradicating *S. aureus* biofilm (García-Salinas et al., 2018; Kostoglou et al., 2020). For instance, in one study, a 240–320 μg/mL concentration of eugenol eradicates 50% of *S. aureus* biofilm (Miladi et al., 2017).

Eugenol can decrease biofilm cell density by killing or inhibiting bacterial growth. When the density of biofilm cells decreases, aggregation and cell-to-cell connections also decrease so that the loosely arranged cells easily separate from each other (Yadav et al., 2015). Since eugenol is a lipophilic molecule, it can disturb the organization of several strata of polysaccharides, fatty acids, and phospholipids, therefore modifying the fluidity and permeability of the cell membrane and finally resulting in cell lysis (Yadav et al., 2015; Wijesinghe et al., 2021). This cell membrane destruction by eugenol has led to the cells’ rough and shrunken appearance, and bacterial cells lose their normal morphology (Yadav et al., 2015). Additionally, it interferes with the intracellular interactions that are crucial for the development of structured biofilms and the establishment of bacterial colonies. The perturbation of these structures can lead to the separation of cells within the biofilm, enabling their facile removal by washing (Yadav et al., 2015). The findings suggested that eugenol’s antibiofilm effect may be attributed to the suppression of cell-to-cell interactions and subsequent cell lysis.

Notably, eugenol exhibited antibiofilm effectiveness against *S. aureus* strains, particularly during the first stages of biofilm development (Kim and Chin, 2023). Biofilm disposal is most effective during the attachment phase of planktonic bacterial cells, which lasts from 0 to 5 h. During this period, at subinhibitory doses, eugenol demonstrated a substantial inhibitory effect on the adhesion ability of *S. aureus* (Apolónio et al., 2014; Kim and Chin, 2023). In addition, in the presence of eugenol, a reduction in the expression of the *sarA* gene was detected (Dunman et al., 2001; El-Far et al., 2021). As previously stated, this gene influences several virulence genes of *S. aureus* and the production of fibronectin, fibrinogen-binding proteins, and toxins. Consequently, it decreases cell adherence to tissues (Dunman et al., 2001; El-Far et al., 2021).

Moreover, the gene expression of *clfA* and *fnbA*, which mediate the initial attachment of bacteria to surfaces, is downregulated by eugenol (Mastoor et al., 2022). Additionally, another study reported that the *Cna* gene’s expression decreases in eugenol’s presence (Mastoor et al., 2022). The collagen-binding protein, Collagen Adhesin (Cna), allows *S. aureus* to adhere to collagen, a key component of the extracellular matrix in host tissues (Patti et al., 1994; Montanaro et al., 1999). All these events lead to a decrease in cell adhesion for biofilm formation. The death of plankton cells and the reduction of cell attachment for biofilm formation negatively affect the next stages of biofilm formation, including biomass production and communication between cells through QS. This property of eugenol can be used to create antimicrobial coatings and polymer films that inhibit the formation of bacterial biofilms on medical and industrial devices (Nostro et al., 2013; Holban et al., 2014; Venkateswaran et al., 2016). For example, one project involved the development of a chitosan-based antimicrobial coating, including embedded mesoporous silica nanoparticles (MSNs) to encapsulate and transport eugenol. The objective was to prevent the formation of biofilms on medical devices. The controlled release of eugenol from the MSNs and coatings occurred sequentially, starting with a low release, then reaching a peak, then decreasing, and finally reaching a plateau. In contrast to coatings lacking eugenol, which had minimal antibacterial properties and still permitted biofilm development after 24 h, the coating containing eugenol not only decreased biofilm formation but also effectively eliminated most of the bacteria attached (Nguyen et al., 2024).

In another study, researchers prepared nanofibers of polyvinylidene difluoride (PVDF) enriched with thymol and eugenol. These nanofibers demonstrated antifouling activity, suppressing biofilm formation by *Escherichia coli* and *S. aureus*, with no aggregation of bacterial cells observed. As a result, this method may address the disadvantage of the short lifespan of nanofibers as a filtration membrane due to clogging by bacteria in water treatment (Bartošová et al., 2022).

As mentioned, eugenol possesses both hydrophilic and hydrophobic properties, facilitating its diffusion in the biofilm matrix (Miladi et al., 2017; Kostoglou et al., 2020). As a result of this diffusion, eugenol can exert its effects on mature biofilms; for instance, the biomass of established biofilms was significantly decreased by the eugenol treatment (Melo et al., 2019; Wijesinghe et al., 2021; Kim and Chin, 2023). Furthermore, the eugenol-treated biofilms substantially reduced the population of live bacteria (Yadav et al., 2015; Melo et al., 2019; Kim and Chin, 2023). In the presence of eugenol, the components of EPS, namely, carbohydrates, protein, and nucleic acids, were significantly decreased (Ni et al., 2022). Also, in one study, DNA/RNA fragments, tryptophan, lipid, carotenoid, and amide of *S. aureus* biofilm in the presence of eugenol were significantly reduced (Kim and Chin, 2023). Furthermore, following eugenol treatment, a significant downregulation occurs in the gene expression of *sarA*, *icaA*, and *icaD* (Yadav et al., 2015; El-Far et al., 2021; Mastoor et al., 2022). As previously mentioned, these genes are involved in synthesizing PIA/PNAG, which is the main component of the EPS matrix in *S. aureus* biofilm. As a result of these alters, the integrity of the biofilm and its protective capacity and stability are reduced, and the biofilm becomes more sensitive to other external agents and antimicrobial substances.

**FIGURE 2:**
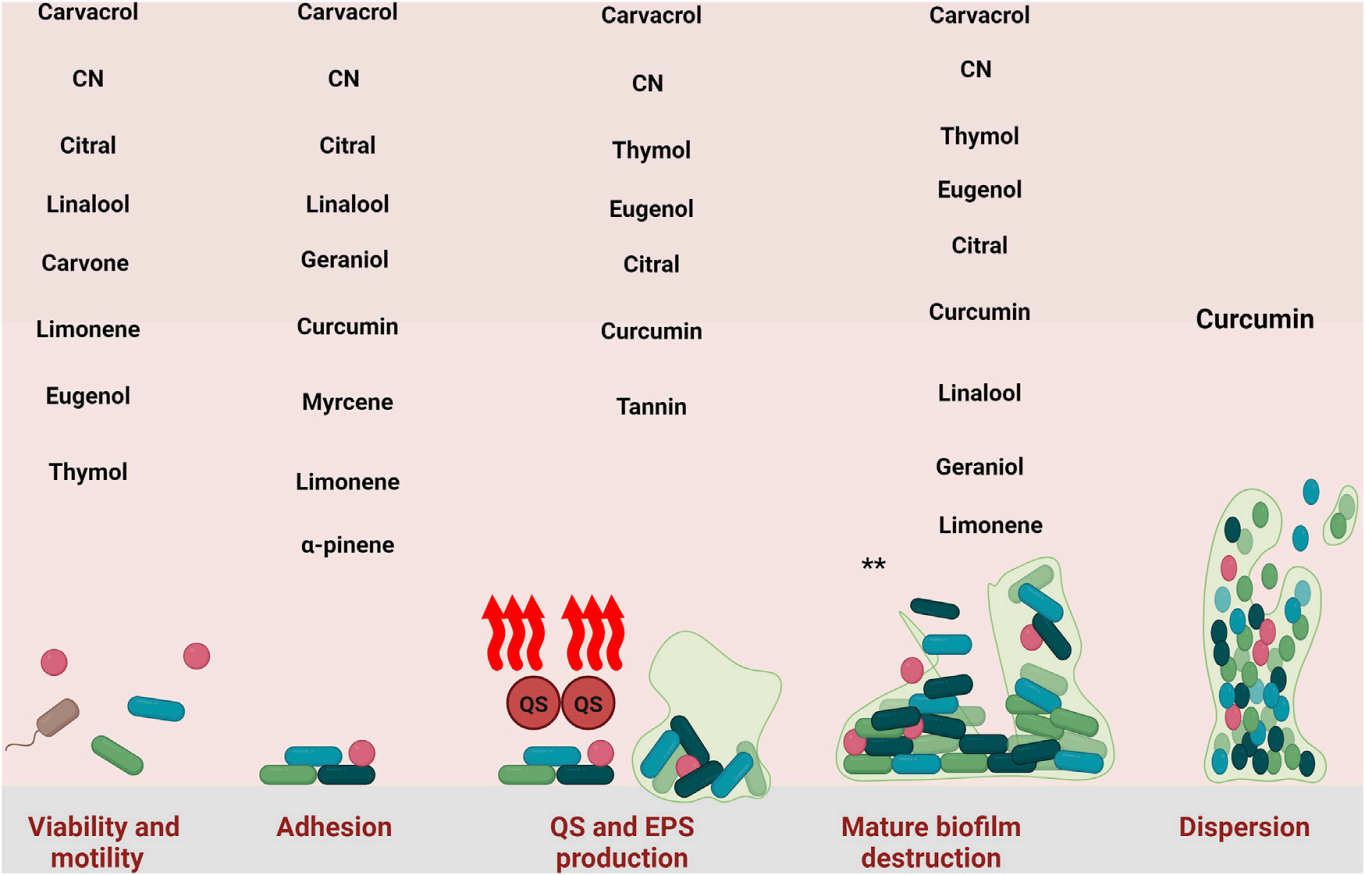
Inhibitory effect of natural compounds against different stage of *S. aureus* biofilm. CN: cinnamaldehyde. *: all of the natural compounds with detrimental effect against mature biofilm are: Carvacrol-thymol- Cinnamaldehyde, Eugenol, Curcumin, Citral, Linalool, Geraniol, Myrcene, Limonene, Myrtenyl Acetate, 1,8-Cineole, α-Pinene, Terpinolene, Linalyl acetate, α-Terpineol, Terpinen-4- ol, Tannin, and Ellagic acid.

In summary, eugenol influences the initial stages of biofilm formation by decreasing the number of viable cells before biofilm development and inhibiting their attachment to surfaces (Figure 2). Also, even after biofilm formation, eugenol can disrupt it. Like other natural compounds, eugenol has some disadvantages, such as low water solubility, poor physicochemical properties, chemical instability, and low bioavailability. These issues can be addressed by combining eugenol with nanoparticles or other drug platforms.

## Quercetin

Quercetin (3,5,7,3ʹ,4ʹ-pentahydroxy flavone) is classified under the flavonol subclass of flavonoids. Quercetin is prevalent in vegetables and fruits, including medicinal herbs like *Hypericum perforatum*, also called Ginkgo (Dengler et al., 2015). Studies have shown the antibacterial and antibiofilm properties of plant extracts containing this compound against *S. aureus* (Sharma et al., 2018; Radojević et al., 2023; de Oliveira et al., 2024). In a study, the MIC value of quercetin against *S. aureus* was found to be 256 μg/mL, and the MBIC value of this compound was determined to be 128 μg/mL (Wu et al., 2023).

Quercetin effectively compromised bacterial cell membranes and walls, resulting in deformation, cytoplasmic leakage, and cellular cavitation while not impacting division and proliferation (Kang et al., 2022; Nguyen and Bhattacharya, 2022). Additionally, this compound demonstrated an inhibitory effect on nucleic acid synthesis and the production of virulence factors in bacterial cells, resulting in a significant antibacterial action (Wang et al., 2018). In the presence of quercetin, both the biofilm thickness and the bacterial count within the biofilm diminished, resulting in a sheet-like dispersion of tiny clusters, with the biofilm manifesting as a single-layer cell aggregation (Lee et al., 2024; Liu et al., 2024).

Studies have shown that quercetin affects cell adhesion for biofilm formation (Kang et al., 2022; D'Arcangelo et al., 2024). Molecular docking and kinetic simulation showed that quercetin could bind ClfB (Kang et al., 2022). In addition, the expression of *fnbA* and *fnbB* altered and significantly downregulated when treated with quercetin (Wu et al., 2023). Quercetin significantly reduced the expression levels of *srtA*, which encodes sortase A enzyme, and the expression of *sigB* (sigma factor B) (Lee et al., 2013; Li Y. et al., 2024). σB is a product of the *sigB* operon and serves as the primary regulator of *S. aureus* response to environmental stress. This factor is crucial in developing bacterial drug resistance, the regulatory expression of virulence-associated genes, and biofilm formation (Peng et al., 2022). σB facilitates the synthesis of many cell surface proteins associated with the initial adherence of biofilms, including FnbA and ClfA. σB enhances the transcription of *fnbA* during early growth and markedly increases the transcription of *clfA* in late growth (Entenza et al., 2005). Therefore, by preventing the expression and function of proteins related to cell adhesion in *S. aureus*, one of the key stages of biofilm formation is affected and disrupted by quercetin.

Quercetin significantly decreased EPS synthesis and secretion (Li Y. et al., 2024; Liu et al., 2024). Additionally, the secretion of eDNA was significantly inhibited with increasing quercetin concentrations (Liu et al., 2024). Further study of the polysaccharide and protein percentages in EPS revealed that quercetin exerted a more pronounced influence on protein secretion (Li Y. et al., 2024).

The transcription levels of extracellular metalloproteinase Aur (aureolysin) and extracellular nuclease Nuc (nuclease) were markedly elevated following quercetin therapy (Liu et al., 2024). Extracellular proteases are crucial in the protein-dependent process. The augmented release of extracellular proteases restricts biofilm development, with metalloproteinase Aur exhibiting the most significant inhibitory effect (Loughran et al., 2014). The concentration of eDNA in the biofilm is modulated by Nuc, which can destroy eDNA and diminish biofilm formation (Kiedrowski et al., 2011). Also, it was reported that the transcription of *aur* and *nuc* is negatively regulated by SarA, while the expression of *sarA* is reduced by quercetin (Liu et al., 2024). Besides, as mentioned earlier, the expression of σ*B* is reduced by quercetin, and on the other hand, σB affects the expression of *sarA* (Bischoff et al., 2001).

In addition to EPS, surface proteins, and eDNA, functional amyloids are one of the components of *S. aureus* biofilm matrix (Schwartz et al., 2012; Karygianni et al., 2020). Biofilm-associated protein (Bap) is a surface-associated protein that assumes an amyloid-like structure under specific environmental circumstances (Di Martino, 2016; Taglialegna et al., 2016). The protein may manifest as amyloid-like clumps on the bacterial surface, facilitating the formation of a robust biofilm structure. These persistent aggregates enable bacteria to cling to diverse surfaces and enhance their resistance to environmental conditions, including antibiotic exposure (Taglialegna et al., 2016). Quercetin inhibits *S. aureus* biofilm development by affecting the production of Bap amyloid-like aggregates without altering Bap expression (Matilla-Cuenca et al., 2020).

Quercetin acts as a quorum-quenching inhibitor. It obstructs bacterial communication by inhibiting the interaction between QS signaling molecules and their receptors, consequently diminishing bacterial motility, proliferation, and metabolic activity (Li Y. et al., 2024). For *S. aureus*, quercetin significantly reduced the expression levels of QS genes (*agrA*) (Lee et al., 2013; Wu et al., 2023; Li Y. et al., 2024). Additionally, as discussed earlier, the expression of *sarA* diminishes due to quercetin, and SarA can affect agr expression. Quercetin functioned as an exogenous inhibitor, suppressing interbacterial communication by modulating the expression of the AGR receptor protein gene in *S. aureus*, thereby managing the expression of downstream genes associated with biofilm formation, bacterial growth and metabolism and effectively diminishing biofilm secretion (Li Y. et al., 2024).

Simply inhibiting QS is insufficient to avert biofilm development. Quercetin may be utilized alongside antibiotics or other antibacterial agents to enhance their antibiofilm effectiveness (Vipin et al., 2020). For example, a study created bi-functional nanoparticles by co-assembling quercetin and copper ions. Copper eradicated bacteria by compromising the cell membrane’s integrity, whereas quercetin interfered with QS processes important for biofilm formation by downregulating the expression of specific genes, effectively inhibiting biofilm development (Cheng et al., 2024).

Like other flavonoids, quercetin exhibits prevalent issues associated with natural bioactive compounds, including inadequate water solubility and diminished bioavailability (Sun et al., 2015). Therefore, various types of drug delivery methods have been studied to overcome this problem, such as hydrogels, nano-micelles, nanoliposomes, and nanoparticles (Akhlaghi and Najafpour-Darzi, 2023; Nain et al., 2023; Yang et al., 2025). For instance, a study concentrated on synthesizing quercetin-encapsulated chitosan sodium alginate nanoparticles (Q-CSNPs). Q-CSNPs employed against *E. coli* and *S. aureus*. The findings indicated that quercetin nanoparticles may suppress or eliminate the bacterial biofilm, regardless of whether treatment occurred before or following biofilm formation. Furthermore, Q-CSNPs demonstrated significant antioxidant ability and notably affected planarians’ oxidative stress (Sun et al., 2024). In another study, hyaluronic acid-modified azithromycin/quercetin micelles (HA-AZI/Qe-M) were produced using thin film hydration. HA-AZI/Qe-M exhibited remarkable antibacterial efficacy *in vitro* and showed the capacity to penetrate deeply into the MRSA biofilm, effectively inhibiting and eradicating it. Moreover, following treatment with HA-AZI/Qe-M, the bacterial count in the thigh muscle tissue of mice was dramatically diminished (Zhang et al., 2024). In the end, the poly (ε-caprolactone)-monomethoxyl poly (ethylene glycol) (PCL-mPEG) micelles, loaded with quercetin and rifampicin (QRMs), were synthesized. The results indicated that the small-sized QRMs may infiltrate the inside of MRSA biofilm to disperse and eliminate it. Subsequently, antibiotics are discharged and concentrated within the acidic biofilm milieu. QRMs may eradicate germs by enhancing bacterial membrane permeability and modifying membrane potential and fluidity. Furthermore, QRMs enhanced drugs’ intracellular and cytoplasmic transport efficiency to epithelial cells (Chen et al., 2022).

In short, quercetin exerts its inhibitory effect on *S. aureus* biofilm by inhibiting bacterial growth, disrupting cell adhesion, reducing the biofilm matrix, altering the expression of genes involved in biofilm formation, and preventing the proper function of QS. Additionally, to enhance its efficiency and reduce its limitations, it can be combined with other drugs and incorporated into drug delivery platforms.

In the end, it is noteworthy that other natural compounds that showed inhibitory effects against *S. aureus* biofilm are presented in Table 3.

**TABLE 3:** Numerous investigations have utilized diverse natural compounds to hinder and break down the biofilm formed by *S. aureus*.

Year of publication	Compounds	Bacterial strains	Source	MIC (conc.)	Outcome	References
2010	Fisetin and esculetin	*Staphylococcus aureus*	Purchased from Sigma-Aldrich	64 and >512 (μg/mL)	Both compounds at a 25 μg/mL concentration significantly reduced biofilm formation	Dürig et al. (2010)
2011	Citral, geraniol and myrcene	*S. aureus, Escherichia coli, Streptococcus agalactiae, Bacillus cereus and*	Compounds of lemongrass oil	0.15–2.5 (µL/mL) Myrcene did not possess antimicrobial activity	These compounds suppressed the primary attachment and biofilm formation of *S. aureus* and destroyed pre-formed biofilms of this bacterium	Aiemsaard et al. (2011)
2011	Linalool and linalyl acetate	*S. aureus*	Purchased from Pollena Aroma	0.19 (v/v %)	These compounds eradicated biofilm of *S. aureus* by up to 90%	Budzyńska et al. (2011)
2011	α-terpineol and terpinen-4- ol	*S. aureus*	Purchased from Pollena Aroma	0.19 (v/v %)	These compounds reduced the biofilm of *S. aureus* by up to 90% at concentrations of 0.38% and 0.19%, respectively	Budzyńska et al. (2011)
2012	Proanthocyanidins	*S. aureus* ATCC 35556 and MRSA	Cranberry extracts	0.08–5 (mg/mL)	The extracts inhibited biofilm production with MBIC between 1.30 and 10 mg/mL	LaPlante et al. (2012)
2013	Ellagic acid	*S. aureus* and MRSA	Purchased from Sigma Aldrich	100 (μg/mL)	Ellagic acid at ½ MIC inhibited biofilm formation and also disrupted pre-formed biofilms	Bakkiyaraj et al. (2013)
2014	Citral and cinnamaldehyde	*S. aureus* and *Salmonella Enteritidis*	Purchased from Aladdin	0.4–0.8 (mg/mL)	The compounds citral and cinnamaldehyde showed substantial inhibition of mixed biofilm formation, while citral was found to decrease the synthesis of AI-2	Zhang et al. (2014)
2014	Eugenol and citral	*S. aureus*, MRSA and *Listeria monocytogenes*	Purchased from Sigma-Aldrich	0.06–0.1 (mg/mL)	These compounds at subinhibitory concentration decreased bacterial adherence	Apolónio et al. (2014)
2014	Genistein, resveratrol, cranberry extract, protocatechuic acid, and p-hydroxybenzoic acid	*S. aureus*	Sigma Chemical Co.	>2000 (µl/mL)	These compounds showed antibiofilm activity	Morán et al. (2014)
2014	Resveratrol	MRSA	Was isolated from natural products	350 (μg/mL)	This compound can destroy QS and the synthesis of capsular polysaccharides and surface proteins	Qin et al. (2014)
2015	Citral and limonene	*S. aureus*	Purchased from PubChem	500–5,000 (µL/L)	The compounds inhibited biofilm formation, and the delay in cell attachment is likely one of the key factors contributing to their effectiveness	Espina et al. (2015)
2015	Sabinene, α-terpinyl acetate, bornyl acetate, limonene	MRSA	Compounds of *Chamaecyparis obtusa* EO	0.1–0.4 (mg/mL)	*C. obtusa* EO inhibited the biofilm formation of MRSA and the expression of virulence factor genes such as *sea*, *agrA*, and *sarA*	Kim et al. (2015)
2015	1.8-Cineole, methyl eugenol, and α-terpinyl acetate	*S. aureus*	Compounds of *Laurus nobilis* L	3.91–15.62 (mg/mL)	*L. nobilis* EO inhibited biofilm up to 70%	Merghni et al. (2015)
2015	Saponin	*S. aureus*	Extract of *Camellia oleifera* seeds	94.5 ± 9.7 (μg/mL)	The saponin showed significant biofilm inhibition and decreased the eDNA.	Ye et al. (2015)
2016	Thymol, menthol and 1,8-cineole	*S. aureus* and MRSA	Purchased from Kemika, Sigma-Aldrich, and Merck	0.250–0.375, 1, and 4–8 (mg/mL) respectively	Thymol and menthol showed acceptable anti-biofilm effects, while 1,8-cineole had weak activity against biofilm	Kifer et al. (2016)
2016	Citral and linalool	*S. aureus*	Purchased from Sigma-Aldrich	0.02 and 0.12 (v/v %)	Citral and linalool inhibited the growth of *S. aureus*, pre-formed biofilms, adhesion, and invasion abilities, and downregulated the virulence genes of this bacterium	Federman et al. (2016)
2016	Darwinolide	MRSA	Isolated from the *Dendrilla membranosa*	132.9 (μM)	Darwinolide displays an IC50 value of 33.2 μM against the biofilm	von Salm et al. (2016)
2017	p-cymene and γ-terpinene	*S. aureus*	Purchased from Sigma-Aldrich and Acros Organics	64–1,024 (µg/mL)	A significant anti-biofilm activity of EO’s was noticed	Miladi et al. (2017)
2017	Citral	*S. aureus*	Purchased from Sigma-Aldrich	0.5 (mg/mL)	Citral had the property of inhibiting biofilm formation and eliminating biofilm cells	Porfírio et al. (2017)
2017	α-Tocopherol	*S. aureus*	Extracted from *Dicranopteris linearis*	>5 (mg/mL)	α-Tocopherol affects the biofilm matrix to disrupt biofilms	Mawang et al. (2017)
2018	α-caryophyllene	*S. aureus* ATCC 25923	Produced by Tokyo Kasei Kogyo Co.	0.507 (mg/mL)	This compound showed good antibacterial and antibiofilm activity	Peng et al. (2018)
2018	Carvacol, γ-terpinene, and α-terpinene	*S. aureus*	Compounds of *Thymus daenensis* EO	0.0625 (μg/mL)	The EO effectively suppressed the development of biofilms by *S. aureus*	Sharifi et al. (2018)
2018	Thymol, γ-terpinene, pcymene and α-terpinene	*S. aureus*	Compounds of *Satureja hortensis* EO	0.125 (μg/mL)	*S. hortensis* EO significantly reduced biofilm biomass	Sharifi et al. (2018)
2018	1,8-cineole	*S. aureus* and MRSA	Purchased from Huiles & Sens	0.048–3.125 (mg/mL)	1,8-cineole displayed the potent efficacy against the development of biofilms and showed anti-quorum sensing activity	Merghni et al. (2018)
2019	Thymoquinone	MRSA and MSSA	Compound of *Nigella sativa* EO	0.0625 (mM)	Thymoquinone effectively reduced the development of bacterial biofilm	Mouwakeh et al. (2019)
2019	Trans-cinnamaldehyde, terpinen-4-ol, and thymol	*S. aureus*, *L. monocytogenes*, *E. coli*, and *Pseudomonas putida*	Purchased from Sigma-Aldrich	0.25–4 (mg/mL)	Significant inhibition of monoculture biofilms was seen with components at ± MIC concentration	Kerekes et al. (2019)
2019	Carvacrol, cymene and thymol	*S. aureus*	Compound of *Satureja montana* EO	0.39–0.78 (mg/mL)	The EO decreased bacterial biofilm formation	Vitanza et al. (2019)
2020	Citral	MRSA	Purchased from Alfa Aesar	200 (µg/mL)	Citral exhibits anti-adherence activity and also regulates the expression of biofilm-associated genes	Valliammai et al. (2020b)
2020	Citral	*S. aureus*, *Candida tropicalis* and *Candida albicans*	Extracted from *Cymbopogon flexuosus* EO	0.0156–0.0313 (v/v %)	Citral decreased the biofilm biomass and cell viability in the biofilm, interfered with the adhesive properties, and disrupted the biofilm matrix	Gao et al. (2020)
2020	Geranyl acetate, γ-terpinene, geraniol, terpinolene, α-pinene, *p*-cimene, and linalool	*S. aureus*	Compounds of *Leptospermum petersonii* EO	1.0 (µg/mL)	The EO caused a 79.88% suppression of the biofilm formed by *S. aureus*	Caputo et al. (2020)
2020	1,8-cineole, trans-sabinene hydrate acetate, globulol, longicyclene, terpinolene, and camphene	*S. aureus*	Compounds of *Eucalyptus gunnii* EO	0.5 (µg/mL)	This EO caused a 60.17% suppression of *S. aureus* biofilm activity.	Caputo et al. (2020)
2020	Eucalyptol and α-pinene	*S. aureus* and *E. coli*	Compounds of rosemary EO	0.5 (mg/mL)	This EO strongly inhibits biofilm formation and induces morphological alterations in biofilms	Liu et al. (2020)
2020	4-terpineol and terpinolene	*S. aureus* and *E. coli*	Compounds of tea tree EO	0.25 (mg/mL)	This EO was shown to be highly detrimental to the developed biofilm and inhibited induced morphological biofilm changes	Liu et al. (2020)
2020	Luteolin	*S. aureus*	Obtained from the Chengdu Pulis	16–64 (µg/mL)	Luteolin destroyed the cell membrane integrity and inhibited biofilm formation	Qian et al. (2020)
2020	Linoleic acid	*S. aureus* DSM 1104	Purchased from Sigma-Aldrich	64 (µg/mL)	It showed biofilm inhibition at sub-MIC concentrations	Yuyama et al. (2020)
2020	Carnosol	clinical strains of *S. aureus*	Purchased from Sigma-Aldrich	32 to 256 (μg/mL)	A reduction in biofilm development and preformed biofilm was observed	Shen et al. (2020)
2020	Tormentic acid	*S. aureus* NCTC 6571	Extract of *Callistemon viminalis*	12.5 (µg/mL)	It detachment of biofilm and decreased eDNA and capsular polysaccharides	Chipenzi et al. (2020)
2021	Citral	MRSA	Purchased from Sigma-Aldrich	5–40 (mg/mL)	Citral decreased the biomass of *S. aureus* and the expression of the *icaA* and *icaD* genes	Oliveira et al. (2021)
2021	β-caryophyllene, d-limonene, γ-terpinene	*S. aureus* and MRSA	Compounds of *Croton piauhiensis* EO	0.15 and 1.25 (v/v %)	The EO showed antibacterial and anti-biofilm effects against *S. aureus*	Do Vale et al. (2021)
2021	α-pinene, linalool, caryophyllene, germacrene D and β-eudesmol	*S. aureus*	Compounds of *Teucrium polium* EO	15–75 (µg/mL)	The EO showed antibiofilm activity and synergistic activity against *S. aureus* strains	Alarjani and Skalicky (2021)
2021	Epigallocatechin gallate	*S. aureus*	Compound of *Camellia sinensis*	7.81–62.5 (μg/mL)	Sub-inhibitory concentrations were able to inhibit biofilm production	Knidel et al. (2021)
2021	Piperine	*S. aureus* MTCC 96	NR	1,000 (µg/mL)	It inhibited the biofilm formation and motility and accumulated ROS in the bacterial cells	Das et al. (2021)
2021	Gallic Acid	*S. aureus*	Purchased from Sigma–Aldrich	≈100–200 (mg/L)	It markedly reduced bacterial growth, biofilm formation, biomass, and EPS levels	Albutti et al. (2021)
2021	Estragole (methyl chavicol or tarragon)	*S. aureus* ATCC 25923	Compound of *Artemisia dracunculus* EO	1.25 (μL/mL)	It showed anti-biofilm and anti-QS activities	Mohammadi Pelarti et al. (2021)
2022	Borneol and citral	*S. aureus* and *Pseudomonas aeruginosa*	Borneol from Guangdong Huaqingyuan, and Citral from Aladdin Biochemical	NR	Citral and borneol exhibited promising anti-biofilm results, and their combination significantly enhanced the anti-biofilm effect	Wang et al. (2022)
2022	Citral and geranial	*S. aureus*, *Staphylococcus epidermidis*, *Klebsiella pneumoniae* and *E. coli*	Compounds of *Backhousia citriodora* EO	6.25–12.50 (µL/mL)	Promising antibacterial and antibiofilm effects were observed against the tested strains	Lim et al. (2022)
2022	Limonene	*S. aureus* and *P. aeruginosa*	Purchased from Sigma Aldrich	20–40 (mL/L)	Limonene works well to inhibit biofilms and destroys mature monospecies and multispecies biofilms	Gambino et al. (2022)
2022	Linalool, Myrtenyl acetate, 1,8-cineole, and α-pinene	*P. aeruginosa, S. aureus, L. monocytogenes, E. coli,* and *Pectobacterium carotovorum*	Derived from *Myrtus communis* L	3–6 (mg/mL)	The EO successfully suppressed the survival of the cells in the biofilm and regulated the metabolic signaling system	Caputo et al. (2022)
2022	Luteolin	*S. aureus* Newman and *agrBDC* mutant	Purchased from Dalian Meilun	64 (µg/mL)	Luteolin inhibits biofilm formation and reduces the transcription of *agrA*	Yuan et al. (2022)
2022	Luteolin	MRSA N315	Purchased from Aladdin	64 (µg/mL)	Luteolin inhibited the biofilm formation and promoted the morphological changes	Sun et al. (2022)
2022	Anthocyanin	*S. aureus*	Obtained from *Lycium ruthenicum Murr*	3.125 (mg/mL)	It can inhibit the biofilm formation and damage the biofilm structure	Dong et al. (2022)
2022	Protocatechuic acid and p-coumaric acid	*S. aureus*	Compounds of *Hericium erinaceus* (HE)	NR	HE exhibited antibiofilm activities with MBIC = 12.5 mg/mL	Darmasiwi et al. (2022)
2023	Linalool	MRSA and VRSA	Purchased from Sigma-Aldrich	0.5–2 (µg/mL)	Linalool showed antimicrobial and antibiofilm activities	Abd El-Hamid et al. (2023)
2023	Carvone and limonene	MRSA	Derived from *Carum carvi* L	0.16 (v/v %)	The EO has a substantial impact on the formation of MRSA biofilm and possesses potent antibacterial properties	Liu et al. (2023)
2023	α-pinene and α-terpineol	MRSA	Compounds of *Pinus koraiensis* EO	2 (mg/mL)	The EO reduced the formation of MRSA biofilms, cell viability, and the expression of *agrA* and *sarA* genes	Kim et al. (2023)
2023	Squalene, γ-terpinene, pinene, p-cymene, caryophyllene oxide	*S. aureus*	Compounds of *Syzygium malaccense* EO	11.7–15 (mg/mL)	The inhibition percentage of biofilm formation by the strains was enhanced by increasing the concentration of EOs	Salem et al. (2023)
2023	Globulol, pinene, p-cymene, and γ-terpinene	*S. aureus*	compounds of *Syzygium samarangense* EO	7.5–11.7 (mg/mL)	The EO showed good dose-dependent antimicrobial and anti-biofilm activity	Salem et al. (2023)
2023	Limonene, β-myrcene, and α-pinene	*S. aureus*	Compound of *Citrus sinensis* EO	2.50–3.125 (mg/mL)	The EO showed potent antibacterial and antibiofilm activity and significantly reduced cell adhesion to the surface	Abdel Samad et al. (2023)
2023	Tannin	MSSA and MRSA	Isolated from *Penthorum chinense* Pursh (TPCP)	156.25 and 312.5 (μg/mL)	TPCP destroyed preformed biofilms, decreased the secretion of exopolysaccharides and extracellular DNA, and regulated the expression of *icaA*, *sarA*, *cidA*, *sigB*, and *agrA*	Qin et al. (2023)
2023	β-sitosterol, phytol, stigmasterol, and lupeol	*S. aureus*	Pulicaria crispa hexane fraction	62.5–125 (µg/mL)	The biofilm formation was reduced by 75.21% at a 250 μg/mL concentration	Abo-Elghiet et al. (2023)
2023	β-ocimene, trans-geraniol, camphor, and eucalyptol	MRSA	*Boesenbergia rotunda* EO (BREO)	4 (mg/mL)	BREO inhibited biofilm formation	Apinundecha et al. (2023)
2023	trans-sabinene hydrate and terpinen-4-ol	MRSA	Compounds of *Origanum majorana* EO	0.3 (mg/mL)	The EO showed a biofilm inhibition rate of 76.6%	Piasecki et al. (2023)
2024	α-pinene	*S. aureus* and MRSA	The main compound of *Euphorbia* EO	50–120 (µL/mL)	This EO exhibited antimicrobial and anti-biofilm activities and inhibited bacterial attachment	Boutoub et al. (2024)
2024	Chlorogenic acid and carnosol	XDR *S. aureus* and *P. aeruginosa*	Chlorogenic acid Purchased from Merck and carnosol identified from *Salvia abrotanoides*	≥1,024 μg/mL	Both compounds effectively inhibited biofilm formation	Sheikhy et al. (2024)
2024	Gallic acid	MRSA	Purchased from Beijing Solarbio	32 (μg/mL)	It significantly inhibited bacterial adhesion and aggregation, affecting the overall structure of the biofilm	Sang et al. (2024)
2024	β-citronellol and geraniol	MRSA	Extracted from *Pelargonium graveolens*	1.56 (µL/mL)	It exhibited anti-adhesive properties and demonstrated the ability to interact with SarA proteins	Elghali et al. (2024)
2024	Epigallocatechin gallate	*S. aureus*	Sourced from Nagara Science	NR	It reduced biofilm formation and the expression of virulence factor-related genes	Oura et al. (2024)

MSSA, methicillin-susceptible *Staphylococcus aureus*; AI-2, autoinducer-2; EO, essential oil; MIC, minimum inhibitory concentration; MRSA, methicillin-resistant *Staphylococcus aureus*; NR, not reported; VRSA, vancomycin-resistant *Staphylococcus aureus*; EPS, extracellular polymeric substances; eDNA, extracellular DNA; IC50, half-maximal inhibitory concentration; MBIC, minimum biofilm inhibitory concentration; ROS, reactive oxygen species.

## Conclusion

Using natural compounds as an anti-biofilm treatment for *S. aureus* demonstrated significant potential for developing new therapeutic approaches. These compounds affect various stages of biofilm formation, including the QS system, biofilm matrix, the attachment of *S. aureus* cells to surfaces and tissues, and the viability of bacteria. Furthermore, these compounds exhibit lower toxicity than traditional antibacterial agents, and because they have multiple purposes, there is less chance of resistance to them occurring. While natural compounds have shown significant potential in laboratory tests, more research is needed to determine their effectiveness *in vivo*. We should not forget that in nature and several other habitats (e.g., the food industry and healthcare), biofilms may be composed of different types of microorganisms that interact with each other in relatively complex ways. Exposure to multispecies biofilms requires investigation. As mentioned, natural compounds have disadvantages such as low bioavailability, insolubility in water, and rapid metabolism and degradation; hence, further studies are needed to optimize their delivery methods. For example, delivery systems based on nanoparticles can improve their penetration into biofilms and increase their stability in complex environments. In addition, their combination with antibiotics and other natural agents can lead to synergistic effects and increase their ability to disrupt the biofilm. Finally, natural compounds–based -photodynamic therapy should also be considered by scientists as a promising approach for managing the biofilm community of *S. aureus*. Therefore, natural compounds are an effective and low-risk option promising to manage *S. aureus* biofilm-related issues.

## Abbreviations

MSCRAMMs, Microbial Surface Component Recognizing Adhesive Matrix Molecules; MRSA, methicillin-resistant *S. aureus*; PIA, polysaccharide intercellular adhesins; NPs, nanoparticles; QS; quorum sensing; EPS, extracellular polymeric substances; eDNA, extracellular DNA; PLA, poly (lactic acid); PS, photosensitizer; ROS, reactive oxygen species; NPs, Nanoparticles; MIC, minimum inhibitory concentration.
